# A Portrait of Breast Cancer in Portugal: The Impact of Racial Disparities

**DOI:** 10.7759/cureus.80047

**Published:** 2025-03-04

**Authors:** Tânia Duarte, Marta D'Orey, José Pereira, Maria H Miranda, Ana Martins

**Affiliations:** 1 Medical Oncology, Unidade Local de Saúde de Lisboa Ocidental, Lisbon, PRT

**Keywords:** black women, breast cancer (bc), breast cancer care, health care disparity, racial disparity

## Abstract

Background

Breast cancer is the most common malignancy among women, with racial disparities in diagnosis, tumor characteristics and outcomes. Black women are disproportionately affected by aggressive subtypes and advanced stage disease, influenced by biological, socioeconomic, and healthcare access factors. While these disparities are well-documented globally, their impact in Portugal remains unknown. The Portuguese healthcare system ensures equal access, including for patients from African Portuguese-speaking countries (PALOPs), who face additional challenges due to the medical evacuation process. This study aims to evaluate differences in breast cancer characteristics and treatment delays between black and white women treated at a Portuguese center.

Materials and methods

A unicentric, retrospective observational study was conducted at a Portuguese center, including 208 women with breast cancer treated between 2022 and 2023. Patients were categorized as black or white, and data were collected on demographic variables, tumor characteristics, stage at diagnosis, and time from symptom onset to treatment initiation. Logistic regression was used to assess the association between the race and breast cancer characteristics. Statistical analyses included Kaplan-Meier survival estimates and Cox proportional hazards models to evaluate survival differences.

Results

Of the 208 patients, 60 were black and 148 were white. The median age at diagnosis was lower for black women (50 years) compared to white women (64 years). Black women were more likely to be diagnosed at stage III or IV (55% vs. 16.2%, p<0.001). Regarding tumor characteristics, black women had a significantly higher prevalence of grade 3 tumors (30% vs. 12.8%, p=0.005), triple-negative breast cancer (TNBC) (31.7% vs. 11.5%, p<0.001) and lower rates of hormone receptor-positive tumors (58.3% vs. 82.4%, p<0.001). No statistically significant difference was found in HER2 expression between groups. The time from symptom onset to treatment initiation was longer for black women (8.5 months vs. 5 months, p<0.001). However, multiple linear regression indicated that being from a PALOP country, was the main predictor of treatment delays (p=0.013), while race alone was not significant (p=0.341). Overall survival analysis was not feasible.

Discussion

The findings of this study showed significant disparities in breast cancer between black and white women. Black women were younger at diagnosis, more frequently presented with advanced stage and aggressive subtypes, and experienced longer treatment delays. The longer time from symptom onset to treatment initiation in black women suggests potential barriers to timely healthcare access, including socioeconomic factors and healthcare system navigation challenges. Further analysis suggests that these delays are primarily driven by patients from PALOPs rather than race itself. These patients face additional systemic barriers, including delays in the medical evacuation process and socioeconomic challenges.

Conclusion

This study highlights the need to address racial disparities in breast cancer care in Portugal. Targeted interventions, policy changes, and improved access to timely care are essential to reducing disparities and improving outcomes.

## Introduction

Breast cancer is the most common cancer in women worldwide, with over 2,296,840 cases annually worldwide [1]. It is the second highest cause of cancer death among women, exceeded only by lung cancer [2].

Over the past two decades, advancements in breast cancer treatment, early detection through mammography screening, and preventive measures targeting risk factors have contributed to a decline in mortality rates [3]. Various social and demographic factors influence these outcomes, including age at diagnosis, geographic location, socioeconomic status, and regional differences in access to screening and treatment [4]. Some differences in incidence, mortality and survival occur by race and ethnicity [2]. An additional and crucial aspect is the impact of racial inequalities [5], which are often less explored in the Portuguese context.

It is well established that survival rates for Black women are lower, when compared to White women [2]. Black and White women have different presentations of breast cancer [2]. Black women are disproportionately affected by more aggressive subtypes of breast cancer, such as triple-negative breast cancer (TNBC), and are more likely to receive a diagnosis at a younger age and at more advanced stages compared to other groups. [3-6]. Despite progress in screening and targeted therapies, Black women continue to experience the highest breast cancer mortality rates, highlighting persistent disparities in outcomes [2, 4-6].

There are many biological and nonbiological factors that contribute to these disparities. Differences are found in the plasma levels of growth factors and hormones, in reproductive factors, and in primary tumor characteristics (including cell cycle proteins, tumor suppressor genes, chromosomal abnormalities and the presence and expression of steroid and growth factor receptors) [7-9]. Nonbiological factors, such as differences in reproductive factors, socioeconomic status, and access to healthcare, also play a role in breast cancer. Many of these factors can be modified to improve patient outcomes [10].

The Portuguese health system provides care equally to a multitude of different ethnicities and races. In Portugal it is estimated that 169,000 people identify as Black race [11]. Significant differences in breast cancer incidence, diagnosis and prognosis have been demonstrated, in other parts of the world, between ethnic and racial groups [4, 12]. In Portugal, the impact of social inequalities and healthcare access in breast cancer between White and Black women is not known.

Also, we have an evacuation process for treatment, between Portugal and the African countries with Portuguese as an official language, six African countries called "PALOPs", the Portuguese acronym for Angola, Cabo Verde, Guinea-Bissau, Equatorial Guinea, Mozambique and São Tomé and Príncipe. The evacuation process for treatment, a mechanism between Portugal and the PALOPs, allows patients to be transferred to Portugal for medical care when a condition is diagnosed in their home country, but the necessary treatment options are unavailable locally. Many of these women face specific challenges in accessing timely and effective breast cancer diagnosis and treatment. These countries are confronted with issues such as low rates of early diagnosis and limited treatment options, due to a lack of adequate infrastructure and specialized professionals. Once a diagnosis is made, the patient can initiate the evacuation process through a proposal submitted to the National Medical Board in their country of origin. However, despite the existence of this evacuation plan, these women also face the compounded challenges of socioeconomic barriers and the strain of navigating medical systems in a foreign country.

In this study, we tried to find the main differences between White and Black women with breast cancer in our center and how they may have a part in prognosis and overall survival in these patients. Investigating these potential disparities is crucial to developing targeted interventions that can address the unique needs of this population, reduce diagnostic and treatment delays, and improve breast cancer survival rates for all women in Portugal.

We performed this real-world data analysis to evaluate the differences between Black and White women with breast cancer. Our goal was to compare the subtypes of breast cancer, the age and stage at diagnosis, as well as the time from the onset of symptoms to the start of treatment between these two groups.

## Materials and methods

We conducted a unicentric, retrospective observational study in one Portuguese center. Patients diagnosed with breast cancer, treated at our center between January 2022 and December 2023, were selected. The inclusion criteria were: age >18 years; female sex; Portuguese nationality or from PALOPs; breast cancer diagnosis confirmed by histology, newly diagnosed early, advanced, or metastatic breast cancer at the time of the analysis; having received treatment (surgery, radiotherapy, systemic treatment and/or best supportive care) at our center between January 2022 and December 2023. Exclusion criteria included: other active cancer; absence of invasive breast carcinoma and medical records lacking information on race.

Data was collected from medical records, including date of birth; age at diagnosis; race; country of origin; performance status; age at menarche; number of children; breastfeeding history; menopausal status; family history of cancer; date of symptom onset; date of breast cancer diagnosis; staging (stage I-IV) at diagnosis; date of beginning of treatment; tumor characteristics such as grade, hormone receptor (HR) status (progesterone and estrogen), Ki-67 proliferation index, and human epidermal growth factor receptor 2 (HER2) status (0, 1+, 2+ with negative in situ hybridization (ISH), 2+ with positive ISH, or 3+) and date of death or last follow-up.

Our aim was to compare the differences between Black and White women with breast cancer regarding the subtypes of breast cancer, the age and stage at diagnosis, as well as the time from the onset of symptoms to the start of treatment between these two groups. Our secondary goal was to compare overall survival (OS) between these two groups. We define OS as the period between diagnosis and either death or the last confirmed visit with the patient still alive.

Statistical analysis

The analysis of demographic characteristics, tumor features, and treatments was performed using descriptive statistics. To assess the association between race and the likelihood of having specific subtypes of breast cancer and a stage at diagnosis, a binary logistic regression analysis was performed, and the odds ratio (OR) was calculated to measure the strength of the association. Additionally, a multiple linear regression analysis was performed to identify significant predictors of treatment delays and an ANOVA was used to compare differences between groups.

Survival curves were estimated using the Kaplan-Meier method and survival of two groups was compared with the pairwise log-rank test. Survival analysis and the time from the onset of symptoms to the start of treatment was conducted using the Cox Proportional Hazards model, with hazard ratio (HR) calculation and 95% confidence intervals.

Statistical analysis was performed using SPSS software v.29 (IBM Inc. Armonk, New York), and all results with a p-value of less than 0.05 were considered statistically significant.

## Results

Between January 2022 and December 2023, 488 women diagnosed with breast cancer were treated at our center, of which 280 were excluded for not meeting the inclusion criteria or for meeting any exclusion criteria. A total of 208 women were eligible for this study. Of the 208 women included, 60 (29%) were Black and 148 (71%) were White women. The median follow-up was 25.5 months (10-47).

The characteristics of the two groups are shown in Table 1. The median age at the time of disease diagnosis in the subgroup of Black women was 50 years vs. 64 years in White women. Of the 60 Black women, 85% were from PALOPs countries, but only 36.7% were in the evacuation program.

**Table 1:** Patient characteristics between Black and White women ECOG - Eastern Cooperative Oncology Group; PALOPs - Portuguese acronym for Angola, Cabo Verde, Guinea-Bissau, Equatorial Guinea, Mozambique, and São Tomé and Príncipe

Patient characteristics	Black women (n=60)	White women (n=148)	p-value
Age at diagnosis in years, median (min-max)	50 (30-87)	64 (38-90)	
Nationality, n (%)			
Portuguese	9 (15)	148 (100)	
PALOPs	51 (85)	0	
Angola	16 (31.4)	0	
Cabo Verde	13 (25.5)	0	
Guinea-Bissau	8 (15.7)	0	
Equatorial Guinea	0	0	
Mozambique	0	0	
São Tomé and Príncipe	14 (27.4)	0	
Evacuation program, n (%)			
Yes	22 (36.7)	0	
No	38 (63.3)	0	
ECOG Performance Status Score, n (%)			
0	54 (90)	129 (87.2)	
1	2 (3.3)	13 (8.8)	
>2	4 (6.7)	6 (4)	
Age at menarche in years, median (min-max)	13 (11-16)	13 (9-16)	
Number of children, median (min-max)	2 (0-5)	2 (0-4)	
Breastfeeding history, n (%)			
Yes	54 (90)	123 (83.1)	
No	6 (10)	25 (16.9)	
Post-menopausal, n (%)	30 (50)	114 (77)	
Pre-menopausal, n (%)	30 (50)	34 (23)	
Family history of cancer, n (%)			
Yes	16 (26.7)	36 (24.3)	
No	44 (73.3)	112 (75.7)	
Stage, n (%)			
I	0	79 (53.4)	
II	27 (45)	45 (30.4)	
III	21 (35)	15 (10.1)	
IV	12 (20)	9 (6.1)	
Stage III or IV, n (%)	33 (55)	24 (16.2)	p<0.001
Time from the onset of symptoms to the start of treatment in months, median (min, max)	8.5 (2-61)	5 (1-19)	p<0.001
Death, n (%)	10 (16.7)	6 (4.1)	

The percentage of Black women diagnosed at stage III or IV was 55% vs. 16.2% in White women. The odds of Black women being diagnosed with stage III or IV were significantly greater than White women (p<0.001; OR 6.315, 95% CI 3.229-12.348). The time from the onset of symptoms to the start of treatment was significantly higher in Black women compared to White women (8.5 months vs. 5 months; p<0.001). However, in the multiple linear regression analysis, race alone was not a significant predictor of delayed treatment (β = -2.4, p=0.341), whereas being from PALOPs countries was significantly associated with longer delays (β = 6.7, p=0.013). Similarly, in the ANOVA, race was not a significant factor (p=0.341), while PALOP status showed a trend toward significance (p=0.13). 

The percentage of pre-menopausal women was higher among Black women (50%) compared to White women (23%).

Table 2 refers to the tumor characteristics. Black women were at a greater proportion diagnosed with grade 3 tumors compared to White women (30% vs. 12.8%; p =0.005; OR 2.887, 95% CI 1.387-6.008). The frequency of TNBC was higher in Black women vs. White women, 31.7% vs. 11.5 respectively (p<0.001; OR 3.571, 95% CI 1.700-7.503). The frequency of HER2 expression between Black women (20%) and White women (15.6%) was not statistically significant (p=0.437; OR 1.359, 95% CI 0.627-2.944). The percentage of ER+ or PR+ tumors in Black women was lower than in White women, 58.3% vs. 82.4%. (p<0.001; OR 0.298, 95% CI 0.153-0.580).

**Table 2:** Tumor characteristics between black and white women CI - confidence interval; HER2 - human epidermal growth factor receptor 2; HR - hormone receptor; OR - odds ratio; TNBC - triple-negative breast cancer

Tumor characteristics	Black women (n=60)	White women (n=148)	OR (95% CI)
Grade, n (%)			
1	6 (10)	41 (27.7)	
2	36 (60)	88 (59.5)	
3	18 (30)	19 (12.8)	2.887 (1.387-6.008)
Subtypes of breast cancer, n (%)			
TNBC	19 (31.7)	17 (11.5)	3.571 (1.700-7.503)
HER2 + / HR -	6 (10)	9 (6.1)	
HER2 + / HR +	6 (10)	14 (9.5)	
HR + / HER2 -	29 (48.3)	108 (72.9)	
Subtype HER2 + (HR +/-), n (%)	12 (20)	23 (15.6)	1.359 (0.627-2.944)
Subtype HR + (HER2 +/-), n (%)	35 (58.3)	122 (82.4)	0.298 (0.153-0.580)
Ki-67, median (min-max)	50.5 (5-90)	20 (5-80)	

Survival analysis

Due to the limited follow-up time and limited number of events (16 deaths among 208 women), the median survival has not yet been reached, when population survival analysis was performed. Thus, data on overall survival was not obtainable in this initial analysis. 

## Discussion

Our study highlights significant differences in age at diagnosis, tumor characteristics, stage at diagnosis and treatment delays between black and white women, aligning with prior research on racial disparities in breast cancer [4-6]. These disparities are not only evident in epidemiological studies but are also supported by emerging preclinical and translational research, which suggests a multifactorial basis for these differences, including genetic, socioeconomic, and healthcare access-related determinants [13-15].

Age at diagnosis and tumor characteristics 

The median age at diagnosis was lower in black women (50 years) than in white women (64 years), consistent with findings that black women are diagnosed at a younger age [3-4, 8]. This trend has been observed in multiple large-scale studies and is partly attributed to genetic predisposition, environmental exposures, and lower rates of routine mammographic screening in black populations [16]. 

One of the most prominent findings was the higher percentage of black women diagnosed at more advanced stages (III or IV) compared to white women, with 55% of black women presenting at these stages versus only 16.2% of white women (p<0.001). These results are consistent with previous studies that have demonstrated a tendency for black women to present with more advanced disease at diagnosis, which may be linked to delays in screening and diagnosis, as well as disparities in healthcare access [4-6, 12]. Socioeconomic factors, such as lower educational attainment, further exacerbate these delays [17].

Triple-negative breast cancer and aggressive tumor subtypes

Studies have shown that black women are more likely to develop aggressive subtypes of breast cancer, such as TNBC, which is typically associated with worse prognosis and lower survival rates [4-5, 18]. In line with these findings, we observed a higher frequency of TNBC in black women compared to white women (31.7% vs. 11.5%, p<0.001). TNBC, which is known for its aggressive nature and lack of targeted therapeutic options, is disproportionately more frequent in black women [4, 6]. This highlights the need for improved prevention strategies, as well as early and more personalized interventions for black women to address this disparity.

Our study further revealed that black women had a higher proportion of grade 3 tumors (30% vs. 12.8%, p=0.005), supporting previous reports that black women are more frequently diagnosed with more aggressive, high-grade tumors, which are known for their rapid growth and poor prognosis [9-10]. Additionally, black women had a lower percentage of ER+ or PR+ tumors (58.3% vs. 82.4% in white women, p<0.001), suggesting that black women are less likely to have hormone receptor-positive tumors, which are generally associated with better outcomes and treatment options [4]. 

Delays in treatment initiation

The time from symptom onset to treatment initiation was also significantly longer for black women (8.5 months vs. 5 months in white women, p<0.001). This delay is concerning, as timely treatment is crucial for improving survival outcomes, particularly in aggressive breast cancer subtypes. However, further analysis suggests that this disparity is primarily driven by patients from PALOPs countries rather than race alone. In the multiple linear regression analysis, PALOP status was significantly associated with longer treatment delays (β = 6.7, p=0.013), whereas race alone was not a significant predictor (β = -2.4, p=0.341). Similarly, ANOVA results showed no significant effect of race (p=0.341), while PALOP status showed a trend towards significance (p=0.13). These findings suggest that the observed delays may be more closely linked to systemic factors affecting PALOP patients, such as socioeconomic barriers, healthcare access limitations, and logistical challenges potentially related to the medical evacuation process. This aligns with previous studies reporting similar patterns of treatment delays among populations facing structural healthcare inequities [2]. Addressing these barriers could be crucial in reducing diagnostic and treatment delays and improving breast cancer outcomes in this vulnerable group.

Implications and future directions 

The implications of these findings highlight the need for multi-level interventions to address racial disparities in breast cancer. Efforts should focus on improving healthcare access, enhancing early detection programs, and developing targeted outreach initiatives for at-risk populations. Health literacy interventions and culturally sensitive patient education programs may help bridge existing gaps in screening and treatment initiation. Additionally, addressing socioeconomic barriers and increasing awareness among healthcare providers about the unique challenges faced by black women are critical steps toward achieving health equity [2, 10].

Furthermore, future research should aim to explore the underlying biological mechanisms that contribute to these disparities. Advances in genomic profiling and single-cell sequencing are shedding light on the molecular underpinnings of racial differences in breast cancer subtypes and treatment response [19-20]. Integrating these biological insights with socioeconomic and healthcare system factors will be crucial in developing more personalized and equitable therapeutic approaches.

Limitations and future research

While our study provides valuable insights into racial disparities in breast cancer in Portugal, it has some limitations. One of the major limitations of this study is the relatively short follow-up period and a small number of events for survival analysis. Further research with longer follow-up times and larger sample sizes is needed. Additionally, the unicentric nature of this study may limit the generalizability of the findings. Since it is based on data from a single institution, the results may not fully reflect the diversity of the broader population. Moreover, while we have identified some important racial disparities in tumor characteristics and treatment delays, further research is needed to investigate the underlying biological mechanisms that contribute to these differences. The role of genetics, as well as hormonal and environmental factors, should be explored to better understand how these disparities manifest at the molecular level.

## Conclusions

This study highlights significant differences in the presentation, treatment delays, and tumor characteristics between black and white women with breast cancer in Portugal. Our findings underscore the importance of addressing racial disparities in breast cancer diagnosis and treatment, as well as the need for targeted interventions to reduce these disparities. Continued efforts are essential to ensure that all women, regardless of race or ethnicity, have access to timely and effective breast cancer care.
